# Tuning Permeability and Transport in Polyelectrolyte Membranes: The Role of Countercations

**DOI:** 10.1021/acs.langmuir.5c01594

**Published:** 2025-07-22

**Authors:** Marta Kolasinska-Sojka, Magdalena Wlodek, Michal Szuwarzynski, Piotr Warszynski

**Affiliations:** † Jerzy Haber Institute of Catalysis and Surface Chemistry, 49559 Polish Academy of Sciences, Niezapominajek 8, PL-30239 Krakow, Poland; ‡ AGH University of Krakow, Academic Centre for Materials and Nanotechnology, al. A. Mickiewicza 30, PL-30059 Krakow, Poland

## Abstract

Polyelectrolyte multilayers (PEMs) are widely utilized in membrane technologies, biosensing, and drug delivery, where precise control over permeability, which refers to the ease of transport through the multilayer, is essential. While the influence of anions on PEMs is well-documented, the role of countercations in regulating transport properties through films remains underexplored. Here, we investigate the effects of sodium (Na^+^) and potassium (K^+^) countercations on the formation, structure, permeability, and transport properties of PAH/PSS and PDADMAC/PSS multilayers. Using a quartz crystal microbalance with dissipation (QCM-D), atomic force microscopy (AFM), cyclic voltammetry (CV), and electrochemical impedance spectroscopy, we demonstrate that K^+^-assembled films exhibit higher mass, denser packing, and significantly reduced permeability compared to Na^+^-assembled films. Extended characterizations reveal selected permeability toward ionic probes and frequency-dependent impedance behavior in K^+^, underscoring the potential of the films as tunable barriers. We further demonstrate their application in a model drug release system, highlighting controlled release profiles influenced by countercation choice. These findings provide insights into cation-mediated tuning of PEM properties, offering a robust strategy for designing advanced materials for separation, sensing, and biomedical applications.

## Graphical abstract

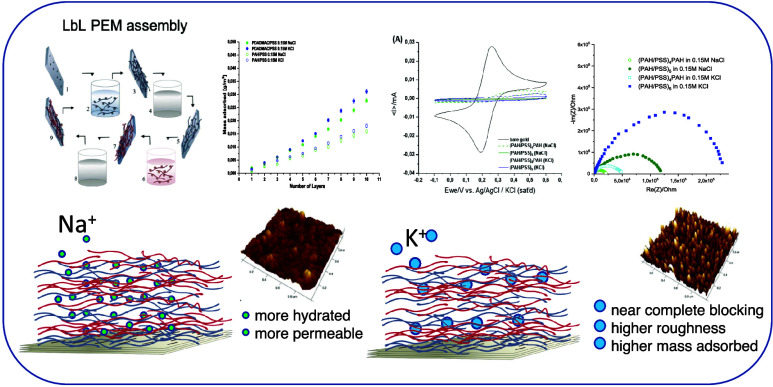

## Introduction

The advancement of functional materials relies on the ability to fabricate nanostructures with a precisely controlled architecture and properties. To this end, considerable attention has been directed toward developing functional nanomaterials via molecular assemblies with tunable composition, structure, and, thus, enhanced properties.^1^ The controlled manipulation of surface characteristics and the directed assembly of various materials, ranging from inorganic nanoparticles to biomolecules, are crucial steps in designing nanostructured materials.^2,3^ Among the systems studied, polyelectrolyte multilayers (PEMs) have emerged as the simple, cost-effective, flexible, and versatile approach for surface modification.^4,5^ The nanoarchitecture of these films, including parameters such as thickness, roughness, surface charge, wetting, swelling, and permeability, can be tailored by adjusting several key factors. These factors include the ionic strength, pH value, types of polyions and electrolytes, deposition time, and number of deposition steps.^6,7^ For example, increasing the electrolyte concentration of the adsorbed solution effectively screens the charge along the polyelectrolyte chain, leading to a coiled chain conformation and resulting in thicker multilayers due to a larger thickness increment per deposition step.^8^ Typically, two primary growth regimes are observed in PEM assembly: linear and exponential.^9^ A well-known example of a linear regime is the PAH/PSS (poly­(allylamine hydrochloride)/poly­(4-styrenesulfonate)) system,^10^ while HA/PLL (hyaluronic acid/poly­(l-lysine))^11,12^ and PGA/PLL (poly­(l-glutamic acid)/poly­(l-lysine))^13,14^ systems commonly exhibit exponential growth. Interestingly, even films that usually grow linearly can switch to exponential behavior when deposition conditions, such as ionic strength, salt type, or temperature, are altered.^15–17^ The role of ions in the assembly process is of paramount importance. The ionic strength affects the multilayer structure, and the specific nature of the ions plays a critical role in determining properties like thickness, permeability, and mechanical characteristics.^17–20^ Ions can be arranged according to the Hofmeister series (also known as the lyotropic series), which categorizes them based on their ability to precipitate proteins.^21^ In this series, chloride often serves as a reference point, with anions to its left being chaotropic (disrupting water structure) and those to its right being cosmotropic (enhancing water structure).^18^ A similar ordering exists for the cations. Studies have highlighted the specific effects of anions on PEM growth. Liu et al.^22^ observed that PEMs formed from PSS and PDADMAC (poly­(sodium 4-styrenesulfonate)/poly­(diallyldimethylammonium chloride)) exhibited nonlinear growth in NaBr, NaClO_3_, and NaCl solutions, while NaF, CH_3_COONa, NaH_2_PO_4_, and Na_2_SO_4_ favored linear growth. Further work by Lutkenhaus and colleagues^23^ revealed that Br^–^ ions, owing to their chaotropic character, had a significantly greater impact on the structural properties of PDADMAC/PSS films than Cl^–^ ions. Similarly, research by Salopek et al.^24^ indicated that the anion type (e.g., NaCl, NaNO_3_, or NaBr) affects the formation process, with nitrate and bromide showing more pronounced influences than chloride. Although extensive research has focused on the effects of Hofmeister anions, the influence of cations has received comparatively less attention. Investigations into the impact of mono- and divalent ions on PEM formation and permeability^25^ have shown that, at the same ionic strength, the presence of Mg^2+^ leads to higher polyelectrolyte adsorption than Na^+^. Moreover, the permeability of the films depended not only on the ionic strength and ion valence in polyion solutions but also on the charge of the electroactive probe used.

In our study, we examine the formation of PEMs in situ using a quartz crystal microbalance with dissipation (QCM-D), with particular emphasis on deposition kinetics and efficiency in the presence of selected electrolytes. While most PEM studies have utilized sodium ions as countercations, potassium ions are predominant in biological systems. Given the wide range of applications of PEMs in biosensors, biomembranes, and drug delivery systems, our work compares multilayers assembled in the presence of potassium versus sodium countercations. Notably, Na^+^, a weak kosmotrope, tends to organize and immobilize water, whereas K^+^, a weak chaotrope, disrupts water organization and enhances its mobility.^26^ Prior research by Cheng et al.^27^ and by Varnai and Zakrzewska^28^ has demonstrated that these cations interact differently with biological macromolecules, including DNA and nucleic acids. Thus, we hypothesize that the nature of the countercation may lead to observable differences in the surface characteristics of PEMs. Accordingly, this paper focuses on investigating the effects of monovalent cations (Na^+^ vs K^+^) on the formation and properties of PDADMAC/PSS and PAH/PSS multilayers. Complementary analyses of the surface topography and permeability of the resulting PEMs were performed using atomic force microscopy (AFM), cyclic voltammetry (CV), and electrochemical impedance spectroscopy (EIS).

## Experimental Section

### Materials

The polyelectrolytes used in this work were poly­(diallyldimethylammonium) chloride (PDADMAC) with a molecular weight in the range of 100–200 kDa, 70 kDa poly­(allylamine hydrochloride) (PAH) as polycations, and 70 kDa polysodium 4-styrenesulfonate (PSS) of as a polyanion. PAH, PSS, and PDADMAC were purchased from Sigma-Aldrich. PDADMAC and PAH were selected as cationic polyelectrolytes due to their contrasting molecular structures and charge characteristics. PDADMAC, a strong polycation with a quaternary ammonium group, exhibits a lower linear charge density, promoting looser, more hydrated films. Conversely, PAH, a weak polycation with primary amine groups, enables pH-dependent charge modulation, resulting in denser films. These properties make them ideal candidates for investigating the role of countercations in modulating PEM assembly and transport properties. For PEM deposition support, silicon wafers with a 100 ± 0.5° orientation (On Semiconductor, Czech Republic) and standard gold/quartz QCM sensors QSX 301 (Q-Sense, Sweden) were used. They were cleaned using a piranha solution, which is a mixture of equal parts concentrated sulfuric acid and hydrogen peroxide (note that this is a highly strong oxidizer and must be handled carefully). Afterward, they were rinsed with Millipore water and soaked for 30 min in hot water at 70 °C. In electrochemical experiments, PEMs were deposited onto gold disk electrodes, which served as the working electrodes. Gold electrodes were polished using aluminum oxide (Al_2_O_3_, φ = 0.05 μm, Buehler, Switzerland). Then, they were cycled in 0.1 M HClO_4_ in the potential range from 0.2 to 1.5 V versus a Ag/AgCl/KCl (saturated) electrode at a scan rate of 100 mV s^–1^.

### Sample Preparation

Deposition of polyelectrolytes by the LbL technique onto silicon wafers or gold electrodes was performed from 0.15 M solutions of sodium chloride or potassium chloride (P.O.Ch, Gliwice, Poland), respectively, with a constant polyelectrolyte concentration of 0.5 g/L. Polyelectrolyte multilayers were assembled under strictly controlled conditions. Each polyelectrolyte layer was adsorbed for 10 min, followed by three rinsing steps (2 min each) in water. The process was repeated until 10 layers of PAH/PSS or PDADMAC/PSS were deposited. The use of consistent parameters across all samples minimized variations in growth conditions and internal structure.

### Quartz Crystal Microbalance

The quartz crystal microbalance with dissipation (QCM-D) control measures the oscillation frequency of a disk-shaped piezoelectric quartz crystal that has metal electrodes on both sides. When stiff films are present, the adsorbate on the electrodes causes a decrease in the resonant frequency (Δ*f*) that is proportional to the adsorbed mass (Δ*m*), following Sauerbrey’s law.

$$\Delta m=-\frac{C\times \Delta f}{n}$$

where Δ*m* is the adsorbed mass, Δ*f* is the shift in the frequency, *C* = 17.7 ng/cm^2^ Hz for sensors used in the present studies, and *n* is the number of the oscillation overtone used for the experiment (*n* = 1, 3, 5, 7, ...). The Sauerbrey relationship is applicable only when the difference between dissipation values for measured overtones does not exceed 10^–6^. In other cases, the viscoelastic models of the film should be used to evaluate its properties, as the dissipation increment (Δ*D*) relates to the viscoelastic properties of adsorbed multilayers. The extended viscoelastic model, implemented in QTools 3 software (Q-Sense AB, Gothenburg, Sweden), was used to interpret the experimental data. Measurements of sequential adsorption of PEs were performed in situ using QCM-D equipment from Q-Sense AB (presently Biolin Scientific). The experiments were conducted at 25 °C.

### Atomic Force Microscopy

The AFM images of “wet” polyelectrolyte films were captured using a Dimension Icon atomic force microscope (Bruker, Santa Barbara, CA) in fluid mode, employing peak force tapping (PFT). Standard silicon cantilevers designed for PFT in fluids (Bruker), with a nominal spring constant of 0.7 N/m and a tip radius of less than 10 nm, were used for these measurements.

### Cyclic Voltammetry and Electrochemical Impedance Spectroscopy

Cyclic voltammetry (CV) and electrochemical impedance spectroscopy (EIS) measurements were conducted with a model SP-300 potentiostat/galvanostat (Biologic, France) equipped with EC-Lab software. The polyelectrolyte multilayers were built on working electrode surfaces using the LbL technique; the Ag/AgCl was the reference electrode, and the platinum sheet was used as a counter electrode. The working electrode was a rotating disk electrode (RDE) with a 3 mm diameter gold disk. Experiments were performed in an equimolar 10^–3^ M solution of potassium hexacyanoferrate­(II) and potassium hexacyanoferrate­(III) with 0.15 M NaCl as the supporting electrolyte. The third cycles of CV were selected to compare all of the results in the potential range of −0.1 to 0.6 V at a scan rate of 50 mV s^–1^.

The EIS measurements were performed by applying a 10 mV AC voltage at frequencies from 10 kHz to 100 mHz at a potential of 220 mV versus the reference electrode. The EIS technique allows analysis of the electrode processes in regard to the effect of electroactive probes as well as the kinetics of electrode reactions and the capacitance of the double layer. Thus, it can be successfully applied to investigate the resistance for the electroactive probes’ transfer through the polyelectrolyte films deposited on the working electrode.^29^ The electrical equivalent circuit (EEC) was introduced to interpret the EIS data.^30^ For an electrode reaction–diffusion system, this circuit is often represented by the well-known Randles equivalent circuit.^30^ That model includes the electrolyte’s high-frequency resistance, ionic charge transfer resistance, double-layer capacitance, and diffusion impedance of electroactive species. An example of such an equivalent circuit model used to analyze EIS spectra for a gold electrode coated with a multilayer polymer film is shown in Figure 1. Before measurements, all solutions were deoxidized by bubbling with laboratory-grade argon (Linde Gas Poland).

**Figure 1:**
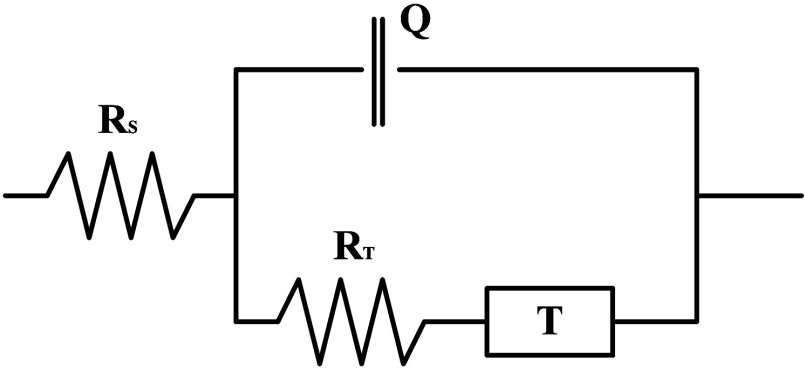
Randles equivalent circuit: T, hyperbolic tangent element; R_T_, impedance of the charge transfer process; Q, constant phase element; R_S_, resistance of the solution.

## Results and Discussion

### Countercation Effect

To investigate the effect of the countercation on the PEM, the build-up process of PAH/PSS and PDADMAC/PSS films was conducted using aqueous solutions of sodium chloride or potassium chloride at an ionic strength of 0.15 M. The mass of the films consisting of 1–10 layers was determined by the QCM-D. The results for the deposition of films in NaCl or KCl are presented in Figure 2. One can see that the build-up process of PAH/PSS and PDADMAC/PSS is nearly linear in both electrolyte solutions. However, when potassium chloride is used as the electrolyte, the mass of the deposited PEM is greater than that in the presence of sodium chloride. This difference is more pronounced in the case of the PDADMAC/PSS film.

**Figure 2:**
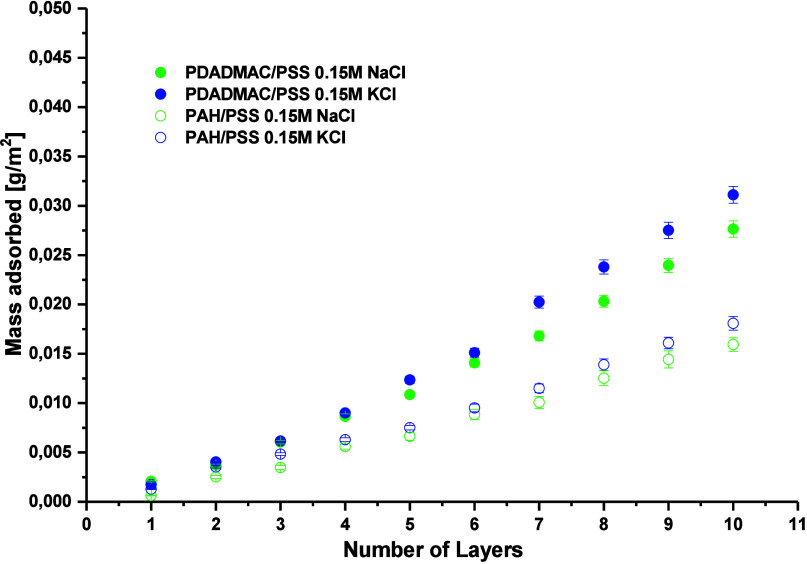
Dependence of the QCM-D-determined mass of PDADMAC/PSS (filled circles) and PAH/PSS (empty circles) multilayers formed from NaCl and KCl solutions, respectively, on the number of polyelectrolyte layers recalculated using the Maxwell viscoelastic model.

The difference in film mass adsorbed depending on the countercation is more pronounced in the case of the PDADMAC/PSS film. It can be explained by a stronger attraction between the potassium ions and sulfonate groups of polyanions, which was demonstrated by the quantum chemical computations by Soldatov et al.^31^ By using DFT (B3LYP/6-31G­(d,p)), they demonstrated that K^+^ forms stronger electrostatic interactions with sulfonate groups of PSS compared to Na^+^, due to its larger ionic radius and weaker hydration shell. This leads to enhanced intrinsic charge compensation, promoting greater chain coiling and increased adsorbed mass in K^+^-assembled films, as observed in our QCM-D results. Additionally, as far as PDADMAC/PSS is concerned, the linear charge density of PDADMAC is much lower than that of PSS, which causes some asymmetry in charge reversal during the build-up process,^32^ resulting, among other things, in the larger amount of polyions adsorbed per layer. However, polyions in such films should be bound more loosely, giving space for other compounds to be transported through them. Analysis of the kinetics of PEM deposition leads to the observation that adsorption from the KCl solution is faster for both studied systems, i.e., PAH/PSS and PDADMAC/PSS, than the process in NaCl. Figure 3 shows a comparison of energy dissipation during adsorption of 10-layer films in NaCl and KCl for (A) PAH/PSS and (B) PDADMAC/PSS. In both studied systems, one can observe a shorter time of deposition of 10-layer films in KCl compared to films adsorbed in the presence of NaCl, and this difference is much greater for PAH/PSS multilayers. The faster deposition kinetics in KCl suggests that K^+^ facilitates quicker polyelectrolyte adsorption possibly by reducing electrostatic repulsion between like-charged groups, allowing faster chain rearrangement. Greater dissipation in KCl indicates more entrapped water and a looser, more hydrated viscoelastic structure.

**Figure 3:**
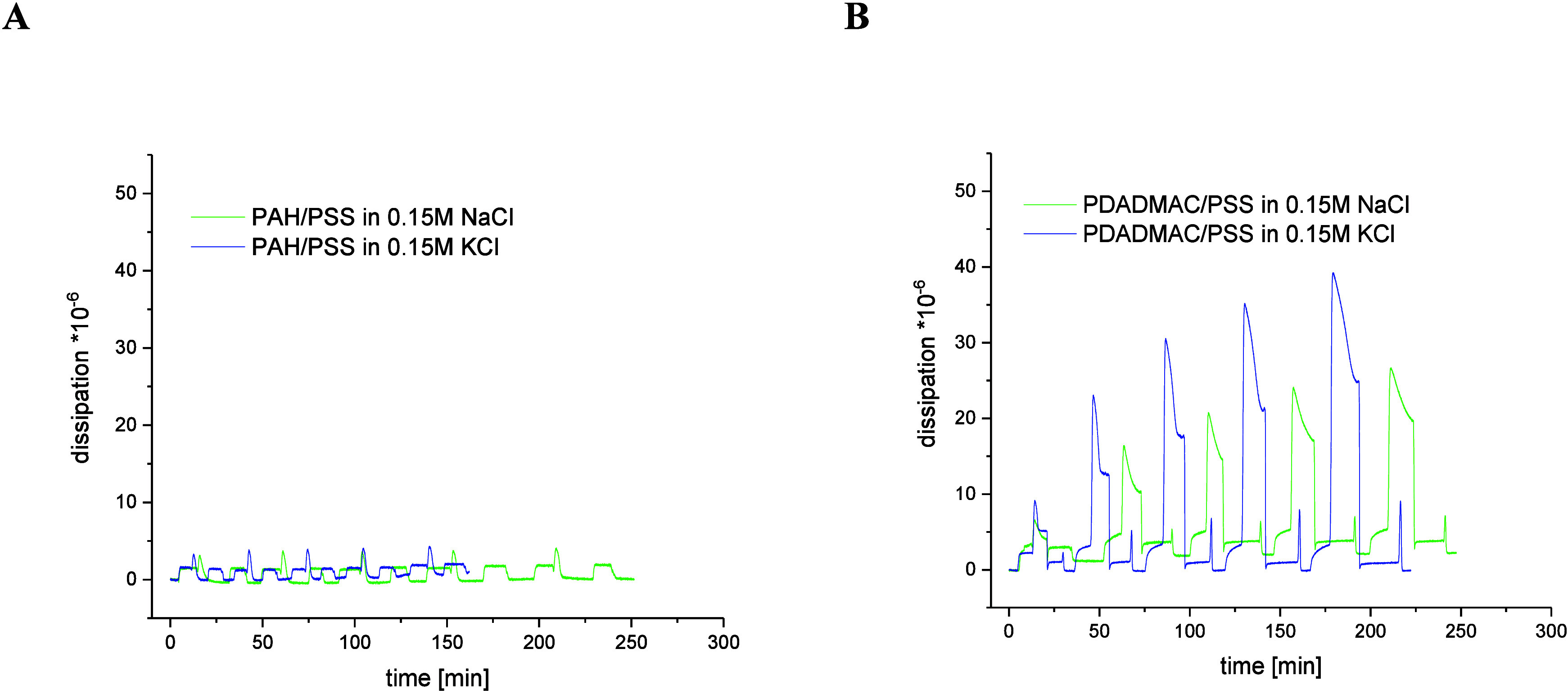
Dependence of energy dissipation over time during the deposition of 10-layer films in 0.15 M NaCl or KCl for (A) PAH/PSS and (B) PDADMAC/PSS.

The surface topography images obtained by AFM for films depending on the countercation are shown in Figure 4. Data for thickness *t* and roughness defined as a root-mean-square height (*R*_sq_) are listed in Table 1. Practically no difference in film thickness was observed between the PEMs formed in both electrolytes. The lack of difference in thickness seems to contradict the QCM-D results showing a mass increase in KCl. However, the QCM-D measures the *in situ* “wet” mass (including hydration water as well as water entrapped in the PEM), while AFM measurements rely on the contact of the AFM tip with the semiwet PEM surface. This suggests that the additional mass in KCl comes from increased hydration and/or looser packing instead from a physical increase in film height. Thus, despite the equal thickness, PDADMAC/PSS films exhibited a higher mass from QCM-D studies. The presence of entrapped water is additionally confirmed by higher energy dissipation in the PDADMAC/PSS films. This interpretation is supported by the comprehensive study of Schönhoff et al.,^33^ which demonstrated that polyelectrolyte multilayers contain significant and variable amounts of water depending on their composition, ionic strength, and assembly conditions. The authors distinguished between different water populations within the films (bound and free water) and showed that hydration could strongly influence the film density and mechanical properties without affecting thickness. According to Schönhoff et al., changes in hydration could also affect viscoelastic behavior and contribute to variations in surface morphology. While AFM images in our study do not suggest large-scale aggregation or morphological instability, we cannot exclude the possibility that differences in internal structure (e.g., density or interdigitation) also play a role.

**Figure 4:**
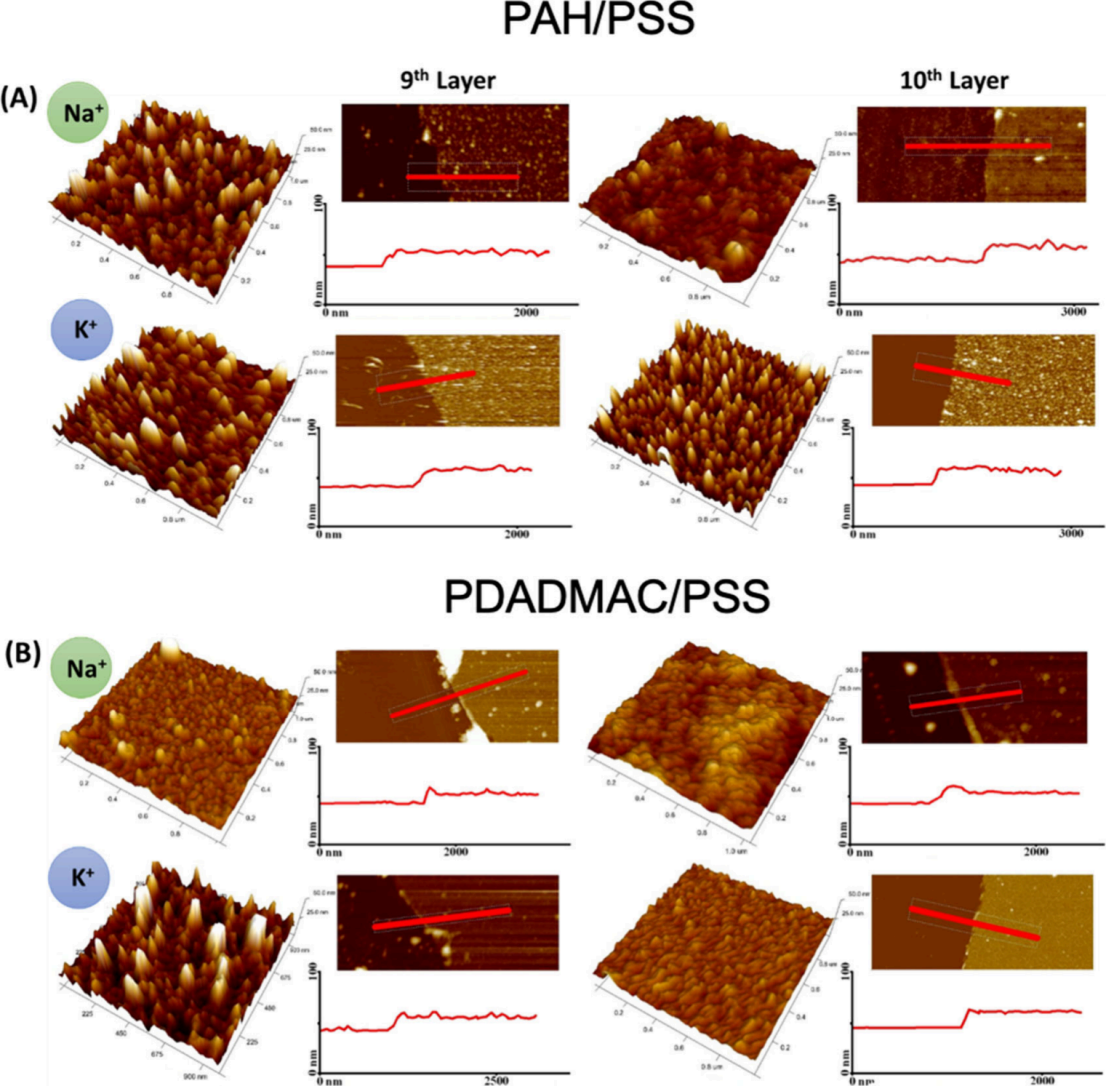
AFM topography images and line profiles of deposited (A) PAH/PSS and (B) PDADMAC/PSS multilayers in the presence of Na^+^ or K^+^.

**Table 1:** AFM Thickness and Roughness Values of Obtained PEMs

	PAH/PSS				PDADMAC/PSS			
	9-layer film		10-layer film		9-layer film		10-layer film	
countercation	*t* (nm)	*R*_sq_ (nm)	*t* (nm)	*R*_sq_ (nm)	*t* (nm)	*R*_sq_ (nm)	*t* (nm)	*R*_sq_ (nm)
Na^+^	12.0 ± 2.8	3.4 ± 0.6	15.7 ± 2.0	3.4 ± 0.8	12.4 ± 2.2	2.9 ± 0.8	15.2 ± 2.3	2.8 ± 1.3
K^+^	12.1 ± 3.1	7.3 ± 1.3	15.3 ± 2.6	5.3 ± 1.5	12.7 ± 3.1	6.9 ± 3.0	14.3 ± 2.1	2.1 ± 0.5

The difference in roughness depending on the countercation was significant in most cases. For 9-layer systems, the roughness was twice as large for multilayers deposited from potassium chloride compared to films in sodium chloride (e.g., 7.3 nm vs 3.4 nm for (PAH/PSS)_4_/PAH and 6.9 nm vs 2.9 nm for (PDADMAC/PSS)_4_/PDADMAC). For 10-layer films, terminated with the PSS polyanion, the difference in roughness is smaller, but still meaningful for (PAH/PSS)_5_ (5.3 nm in KCl vs 3.4 nm in NaCl) and negligible for (PDADMAC/PSS)_5_ (2.1 nm vs 2.8 nm). This fact can be attributed to the difference in energy of the compensating cation exchange at the PSS chain compared to the cationic group of the polycation. The Na^+^ ions are easier to exchange, and the adsorption of the polycation is more uniform. In other words, K^+^ induces a less uniform polycation adsorption, possibly due to stronger PSS^–^–K^+^ interactions that hinder the polycation’s ability to spread evenly. For PSS-terminated films, the difference in roughness decreases, proving that PSS adsorption is less susceptible to the cation type in a supporting electrolyte than those terminated with a polycation.

These observations are strongly supported by the literature. In particular, Schlenoff and co-workers have shown that counterion hydration governs the balance between intrinsic and extrinsic charge compensation in PEMs.^7,35^ Less hydrated ions such as K^+^ are more readily displaced from the film interior, promoting intrinsic compensation via direct interpolymer binding. This mechanism enhances chain coiling and film compaction while increasing water uptake, a phenomenon previously described by Schönhoff et al.^33^ and clearly consistent with our findings.

To gain more detailed insight into the PEM structure, our studies were expanded to include electrochemical measurements, which enabled the determination of the permeability of multilayers and effective diffusion coefficients of electroactive probes. For the permeability determination, cyclic voltammetry was applied. Voltammetric curves obtained for the bare electrode and those covered with PAH/PSS and PDADMAC/PSS films consisting of 9 and 10 PE layers are depicted in Figure 5. The extent of redox current attenuation varies depending on the polyelectrolyte pair and the type of salt used in PEM formation. Notably, PAH/PSS films exhibited lower permeability compared to PDADMAC/PSS films. Thus, in agreement with our previous studies,^29^ films that become thicker with the number of deposited polyelectrolyte layers (PDADMAC/PSS) are more permeable, likely due to increased film hydration and a looser structure. Additionally, the results clearly show a significant difference in redox current blocking (reduced electrochemical probe access) by films formed in the presence of K^+^ ions compared to Na^+^ ions.

**Figure 5:**
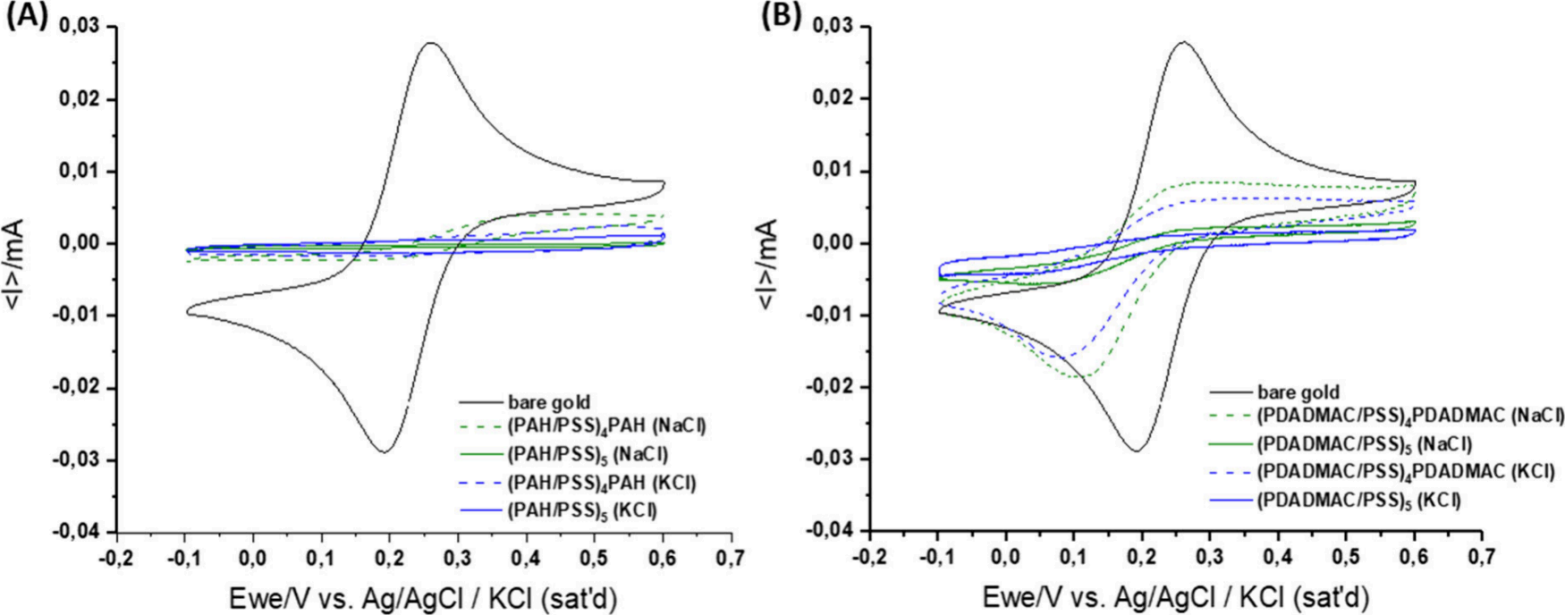
Comparison of voltammetric curves for bare gold electrode and the electrode covered with (A) PAH/PSS and (B) PDADMAC/PSS films with 9 and 10 layers deposited at 0.15 M NaCl (green line) and KCl (blue line).

To extend the analysis, the normalized current reduction factor (*N*)^34^ was calculated according to

$$\Delta N=\left(I_{\mathrm{bare}}-I_{L}\right)/\left(I_{\mathrm{bare}}-I_{E}\right)$$

where *I*_bare_ is the cathodic peak current at the bare gold electrode in the solution of the electroactive probe solution, here measured as −0.02908, *I*_L_ is the cathodic peak current at an electrode with a polyelectrolyte multilayer in a similar solution of the electroactive probe, and *I*_E_ is the cathodic current at the electrode with a polyelectrolyte multilayer in a solution of the pure base electrolyte at the potential of the cathodic peak of the bare electrode, measured as 0.002 mA. Calculated data are listed in Table 2.

**Table 2:** Cathodic Peak Current (*I*_L_) at an Electrode with a Polyelectrolyte Multilayer and Normalized Current Reduction Factors (Δ*N*) Calculated for 9- and 10-Layer Films

	PAH/PSS		PDADMAC/PSS	
	*I*_L_ (mA)	Δ*N*	*I*_L_ (mA)	Δ*N*
9-Layer Film (+terminated)				
Na^+^	–0.003	0.929	–0.0190	0.371
K^+^	–0.002	0.964	–0.0162	0.476
10-Layer Film (−terminated)				
Na^+^	–0.001	0.993	–0.0056	0.867
K^+^	0.0015	0.982	–0.0045	0.907

The value of Δ*N* represents the degree of electrochemical blocking by the polyelectrolyte multilayers, with Δ*N* = 0 for a fully permeable film and Δ*N* = 1 for a completely impermeable one.

One can see that normalized current reduction factors for PAH/PSS films are higher than those for PDADMAC/PSS films. They are all above 0.9, with precise values for 9-layer PAH/PSS in Na^+^ of 0.929 indicating strong blocking against the electroactive probe and in K^+^ of 0.964 showing even stronger blocking. On the contrary, 9-layer PDADMAC/PSS films are characterized by much higher permeability and thus lower blocking ability against the electroactive agent, having Δ*N* values of 0.371 for multilayers in the presence of Na^+^ and 0.476 for films in the presence of K^+^. In the case of negatively terminated 10-layer films of PAH/PSS, nearly complete blocking is observed for multilayers with either countercation, while for PDADMAC/PSS, those values are correspondingly smaller, being equal to 0.867 for films deposited with Na^+^ as the counteraction and 0.907 for multilayers with K^+^.

Electrochemical impedance spectroscopy (EIS) provided complementary insights into the internal structure and ionic transport properties within the multilayers. EIS data in the form of Nyquist plots for gold electrodes covered with films analogous to those for voltammetric measurements are depicted in Figure 6. They show distinct semicircles at high frequencies, which reflect charge transfer resistance at the PEM–electrolyte interface. The significantly larger semicircles observed for potassium-containing films clearly indicate greater charge transfer resistance, consistent with denser multilayer packing, as evidenced by the QCM-D and AFM and stronger polymer–polymer interactions. The nearly vertical low-frequency tail for both types of polyelectrolyte films in the presence of potassium ions indicates capacitive behavior, suggesting that the films are effective barriers to ion diffusion. The Randles equivalent circuit (Figure 1) used to model the EIS data includes *R*_s_ (solution resistance), *R*_ct_ (charge transfer resistance), *C*_dl_ (double-layer capacitance), and *W* (Warburg element). However, the absence of a clear Warburg region (45° line) in the plots suggests that diffusion through the film is minimal. Thus, the EIS results corroborate the CV findings. A higher charge transfer resistance corresponds to a higher current reduction factor Δ*N*, and both indicate that K^+^ enhances blocking more than Na^+^, especially in PDADMAC/PSS. The EIS data provide a frequency-dependent view, revealing that the blocking effect persists across a wide frequency range, making these films suitable for applications requiring stable barrier properties.

**Figure 6:**
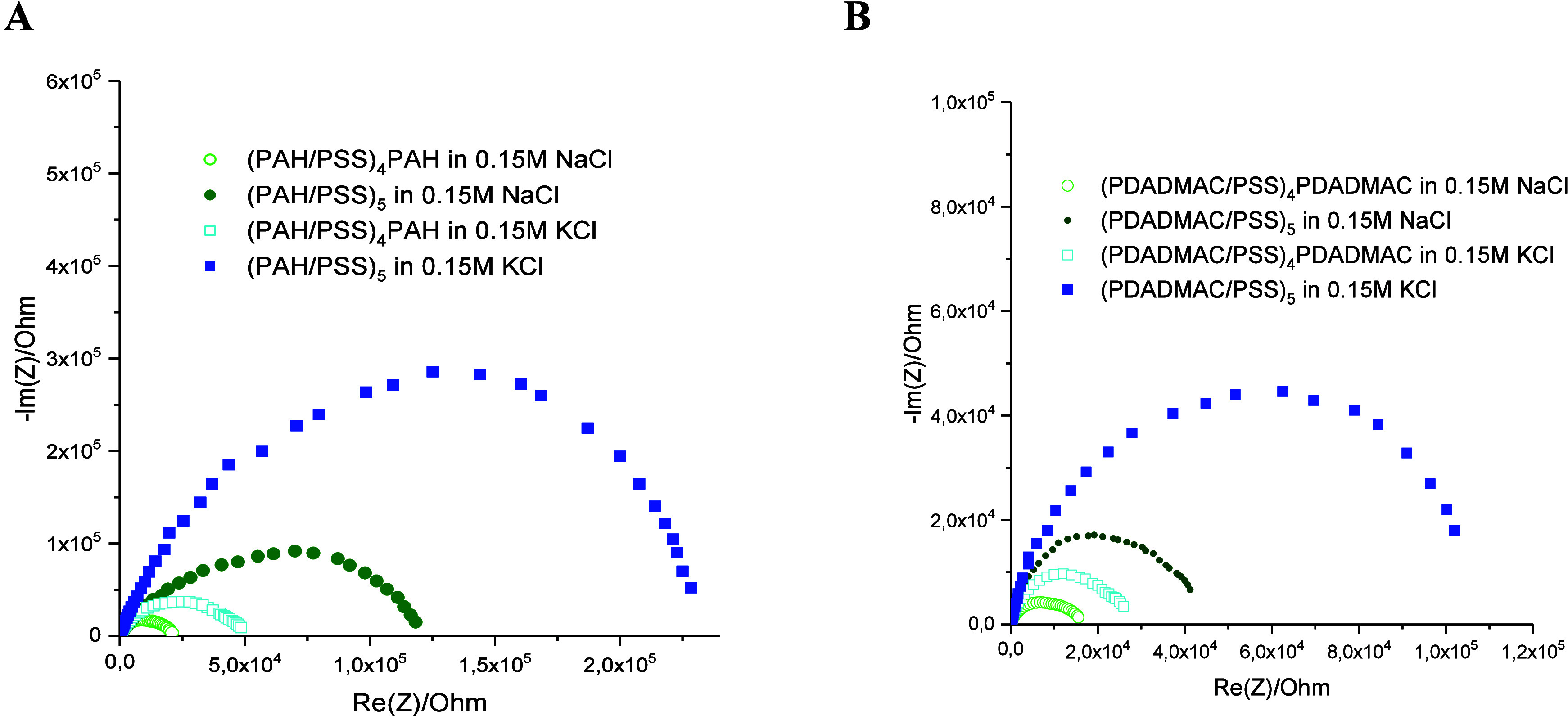
Impedance spectra of Fe­(CN)_6_^4–^/Fe­(CN)_6_^3–^ in 0.15 M NaCl of gold electrodes covered with (A) PAH/PSS and (B) PDADMAC/PSS films with 9 and 10 layers deposited in the presence of NaCl (green points) and KCl (blue points).

The effective diffusion coefficients were determined based on electrochemical impedance spectroscopy. They were calculated for PEM-modified gold electrodes using the linear plot of impedance magnitude versus ω – 1/2 in the low-frequency region at a potential of 220 mV versus the reference electrode, and they are listed in Table 3. All diffusion coefficients for PEM-modified electrodes are much smaller compared to bare gold electrodes, which means that the thickness and porosity of the film affect the transport of the redox species through the PEM membrane. Comparing *D*_eff_ depending on the countercation, one can see that for all studied cases, films adsorbed in the presence of potassium ions are less permeable to redox probes than analogous multilayers prepared with sodium ions.

**Table 3:** Effective Diffusion Coefficients for PEMs Deposited at 0.15 M NaCl and KCl

	effective diffusion coefficient *D*_eff_ (cm^2^ s^–1^)			
	PAH/PSS		PDADMAC/PSS	
countercation	9-layer film	10-layer film	9-layer film	10-layer film
Na^+^	1.94 × 10^–7^	3.14 × 10^–9^	1.57 × 10^–6^	1.67 × 10^–7^
K^+^	1.34 × 10^–8^	2.27 × 10^–9^	9.24 × 10^–7^	1.48 × 10^–8^
	bare gold electrode, 6.30 × 10^–6^			

Such a broad approach allowed us to describe the film structure and density of polyions in a multilayer. The cyclic voltammetry experiments demonstrated a significant difference in the redox current blocking by films formed in the presence of K^+^ and Na^+^ ions. The effect was more pronounced in the case of PDADMAC/PSS films, which turned out to be more permeable for the selected electroactive probe than PAH/PSS films. The PDADMAC/PSS films with the same number of layers formed in a KCl solution attenuated redox current stronger than one deposited in a NaCl solution. This resulted from the tighter structure (higher density due to the coiling of polyions in the presence of potassium ions).

The effective diffusion coefficients calculated for PEM-modified gold electrodes, using EIS, confirmed the CV results, showing that for all studied cases, films adsorbed in the presence of potassium ions were less permeable to the redox probe than analogous multilayers prepared with sodium ions. Although the effective diffusion coefficients were influenced by the transport of ions of the supporting electrolyte through the PEM film, *D*_eff_ gave us general insight into membrane permeability.

## Conclusions

This study demonstrates that the identity of the countercation (Na^+^ vs K^+^) used during polyelectrolyte deposition plays a critical role in determining the formation, structure, and transport properties of PAH/PSS and PDADMAC/PSS multilayers. QCM-D measurements revealed that films assembled in the presence of K^+^ exhibit significantly higher adsorbed mass and faster deposition rates, particularly for PDADMAC/PSS. This is attributed to stronger K^+^–PSS interactions that enhance polyelectrolyte chain coiling and promote water retention. Interestingly, AFM measurements showed that despite these mass differences, the film thickness remained nearly constant, while surface roughness increased, especially in polycation-terminated films. These findings point to hydration-related mass differences and suggest changes in the internal packing rather than physical film expansion. While AFM revealed no clear morphological instability, the higher roughness in K^+^-assembled films may reflect less uniform adsorption and greater viscoelasticity, in line with QCM-D dissipation data.

These observations are strongly supported by the literature. Less hydrated ions like K^+^ are more readily displaced from the film interior, promoting intrinsic compensation via direct interpolymer binding. This mechanism enhances chain coiling and film compaction while increasing water uptake, a phenomenon previously described by Schönhoff et al.^33^ and clearly consistent with our findings. As a result, K^+^-assembled films exhibit not only higher hydration but also reduced permeability and ion diffusion, as confirmed by CV and EIS. In particular, EIS Nyquist plots showed higher charge transfer resistance and capacitive behavior for K^+^-based systems, especially in PDADMAC/PSS films, which are otherwise more permeable due to their looser intrinsic structure.

Collectively, these results underscore the ability to tune the permeability and selectivity of polyelectrolyte multilayers through specific ion effects. For example, K^+^-assembled PAH/PSS films, which exhibit nearly complete blocking, are promising for selective filtration and barrier applications. Conversely, the higher permeability of Na^+^-assembled PDADMAC/PSS films may be advantageous in drug delivery systems requiring controlled release. The synergistic effects of polycation type (PAH vs PDADMAC) and countercation selection (Na^+^ vs K^+^) offer a versatile strategy for tailoring multilayer performance, reinforcing the importance of careful electrolyte and polyion selection in PEM design for targeted applications.
